# Progressive Behavioral Impairment and Region-Specific Monoaminergic Alterations in a Rat Model of Delayed Neuropsychiatric Sequelae After Acute Carbon Monoxide Poisoning

**DOI:** 10.3390/brainsci16060647

**Published:** 2026-06-18

**Authors:** Sungwoo Choi, Heewon Yang, Yuri Kang, Minji Lee, Doo Hwan Lee, Sangchun Choi

**Affiliations:** 1 Emergency Department, Soonchunhyang University College of Medicine, Soonchunhyang University Bucheon Hospital, Bucheon 14584, Republic of Korea; 121791@schmc.ac.kr; 2 Hyangseol Clinical Laboratory, Soonchunhyang University Bucheon Hospital, Bucheon 14584, Republic of Korea; 59club@daum.net (Y.K.); a0016735@schmc.ac.kr (M.L.); a0017476@schmc.ac.kr (D.H.L.); 3 Emergency Department, Ajou University School of Medicine, Suwon 16499, Republic of Korea; speedheewon@gmail.com; 4 Research Department, IBEX Medical Systems, Wonju 26348, Republic of Korea; 5 Emergency Department, Saint Carollo Hospital, Suncheon 57931, Republic of Korea

**Keywords:** carbon monoxide poisoning, delayed neuropsychiatric sequelae, dopamine, cognitive impairment, neuroinflammation, rat model, monoaminergic dysfunction

## Abstract

**Highlights:**

**What are the main findings?**

- Acute carbon monoxide (CO) exposure induced reproducible delayed neurobehavioral deficits involving cognitive, locomotor, and sensorimotor domains in rats.
- Region-specific monoaminergic alterations, particularly dopaminergic depletion in the cortex and basal ganglia, were associated with persistent histopathological abnormalities during the delayed phase after exposure.

**What are the implications of the main findings?**

- The findings support the concept that delayed neuropsychiatric sequelae (DNS) represent an evolving circuit-level neuropathological process rather than a transient hypoxic insult alone.
- Delayed dopaminergic dysfunction may contribute to persistent neurobehavioral impairment and represent a potential therapeutic target following acute CO poisoning.

**Abstract:**

Background: Acute carbon monoxide (CO) poisoning can cause delayed neuropsychiatric sequelae (DNS) after a latent period, yet its pathophysiology remains poorly understood because of the lack of reproducible experimental models. Methods: We established a rat model of DNS using acute CO poisoning (6500 ppm for 25 min). Behavioral assessments evaluated cognition, locomotion, sensorimotor function, exploratory behavior, and reward responsiveness. Histopathological analyses assessed brain injury, and regional monoamine concentrations were quantified using high-performance liquid chromatography. Results: CO-exposed rats developed delayed and progressive behavioral abnormalities, including impaired spatial working memory, reduced locomotor activity, sensorimotor dysfunction, and diminished exploratory behavior. At 4 weeks, CO-exposed rats showed reduced Y-maze alternation (49.3% vs. 72.2%, *p* < 0.0001), complete loss of tape-removal success (0% vs. 100%, *p* < 0.001), reduced digging behavior (10.1 ± 6.9 vs. 27.4 ± 3.9, *p* < 0.01), and decreased locomotor activity (330.5 ± 172.1 vs. 730.5 ± 139.5 cm, *p* < 0.01). In contrast, olfactory discrimination, sucrose preference, and grip strength were preserved. Histopathology demonstrated persistent neuronal and inflammatory alterations. Dopamine concentrations were significantly reduced in the cortex and basal ganglia, whereas thalamic serotonin levels were increased following CO poisoning. Conclusion: Acute CO poisoning induces a reproducible DNS characterized by progressive behavioral impairment, persistent histopathological abnormalities, and regional monoaminergic dysregulation. These findings support the concept that DNS is an evolving neuropathological process and identify dopaminergic pathways as potential therapeutic targets.

## 1. Introduction

Acute carbon monoxide (CO) poisoning remains a major cause of toxic exposure worldwide and is associated with substantial morbidity and mortality [1,2,3]. Beyond acute toxicity, many survivors develop delayed neuropsychiatric sequelae (DNS), a debilitating syndrome that emerges after a latent interval and is characterized by cognitive impairment, neuropsychiatric disturbances, and sensorimotor dysfunction [2,4,5]. DNS may occur in up to 60% of survivors, and its manifestations often persist long term, leading to significant functional disability and socioeconomic burden [3,6,7,8].

Despite its clinical importance, the pathophysiology of DNS remains incompletely understood [9,10,11]. Multiple mechanisms—including delayed demyelination, neuroinflammation, oxidative stress, mitochondrial dysfunction, and excitotoxicity—have been proposed, suggesting a complex and multifactorial process without a unifying framework [3,7,12,13,14,15]. Notably, the role of monoaminergic dysfunction, particularly dopaminergic signaling, in delayed neurobehavioral impairment has not been clearly established, despite its central involvement in cognition, motivation, and motor control [16,17,18,19,20,21,22].

Among these mechanisms, dopaminergic dysfunction has received increasing attention because dopamine plays a critical role in cognition, motivation, motor control, and executive function. Experimental studies have demonstrated that carbon monoxide exposure can alter dopamine release, metabolism, and oxidative processing, potentially contributing to delayed neurobehavioral impairment. Nevertheless, the relationship between region-specific monoaminergic alterations and the development of DNS remains poorly characterized.

Progress in elucidating DNS mechanisms has been further hindered by the lack of reproducible animal models that faithfully recapitulate its delayed onset and multidomain features. Most existing models focus on acute toxicity or mortality and provide limited insight into the delayed behavioral and neurochemical changes that define DNS [23,24,25].

Therefore, the present study aimed to establish a reproducible rat model of DNS following severe acute CO poisoning and to systematically characterize delayed behavioral, histopathological, and neurochemical alterations. We specifically investigated region-specific changes in monoaminergic neurotransmitters, with a particular focus on dopaminergic dysfunction, and examined their association with neuroinflammation and delayed functional impairment. We hypothesized that delayed-phase dopaminergic dysfunction contributes to the pathophysiology of DNS and may represent a key neurochemical mechanism underlying delayed neurobehavioral deficits after CO poisoning.

## 2. Materials and Methods

### 2.1. Study Design and Animals

This study employed a controlled experimental design comparing two groups: a control group exposed to room air and a carbon monoxide (CO)-exposed group subjected to acute CO exposure. The experimental unit for all analyses was an individual animal.

Male Sprague–Dawley rats (280–320 g, 6 weeks old) were obtained from Samtako Bio Korea Co., Ltd. (Osan, Republic of Korea). Animals were housed in standard polycarbonate cages with bedding under specific pathogen-free conditions with controlled temperature (20–24 °C), humidity (40–60%), and a 12 h light/dark cycle. Food and water were provided ad libitum. All animals were acclimated for 7 days prior to experimentation.

All procedures were approved by the Institutional Animal Care and Use Committee (IACUC) of Soonchunhyang University Bucheon Hospital (Approval No. SCHBCA 2023-10) and conducted in accordance with the National Institutes of Health Guide for the Care and Use of Laboratory Animals.

**Figure 1:**
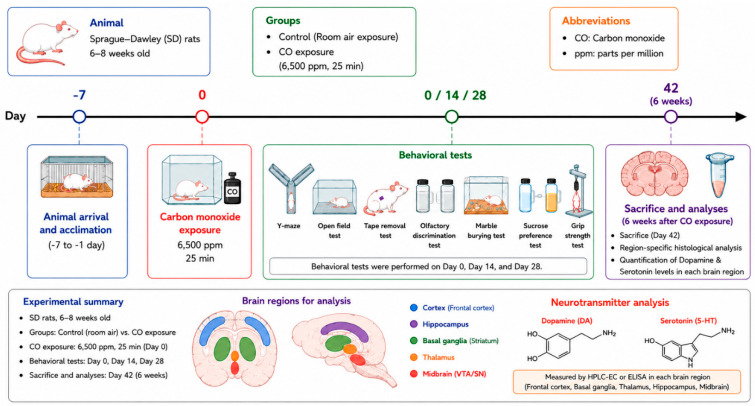
Experimental timeline and procedures.

An overview of the experimental design and study timeline is shown in Figure 1.

### 2.2. Carbon Monoxide Exposure

Acute CO poisoning was induced using a sealed exposure chamber with controlled gas delivery. Rats were exposed to CO at 6500 ppm for 25 min, a condition optimized to induce acute toxicity while ensuring survival and allowing development of DNS.

The exposure condition (6500 ppm for 25 min) was selected based on preliminary dose-finding experiments and previous animal studies demonstrating reliable induction of DNS while maintaining a high survival rate. Although direct comparison between animal exposure and human poisoning is difficult, this model was designed to reproduce severe acute CO poisoning associated with a high risk of DNS development and to provide a translational platform for mechanistic investigation.

Only a small number of animals required brief supplemental oxygen administration during the immediate recovery period after CO exposure. Oxygen supplementation was provided according to predefined criteria and standardized across all animals to minimize potential confounding effects.

CO concentrations were continuously monitored using a calibrated electrochemical sensor (testo 300LL, Testo Korea, Ltd., Seoul, Republic of Korea). Following exposure, animals were returned to room air and monitored for recovery.

Control animals underwent identical procedures, including chamber placement, without CO exposure.

Animals were randomly allocated to control and CO-exposed groups using a simple randomization procedure before exposure. Cage position and behavioral testing order were counterbalanced to minimize potential environmental confounding effects.

Animals were monitored daily for signs of distress, including reduced activity, abnormal posture, respiratory difficulty, and impaired feeding behavior. Supplemental oxygen and supportive care were provided as needed after CO exposure. Humane endpoints included severe respiratory distress, inability to ambulate, or persistent moribund condition requiring euthanasia.

### 2.3. Behavioral Assessments

Behavioral assessments were performed to evaluate multidomain neuropsychiatric dysfunction relevant to DNS, including cognition, locomotor activity, sensorimotor integration, anxiety-related behavior, and reward-related responses. Testing was conducted at baseline (pre-exposure), 2 weeks, and 4 weeks after carbon monoxide exposure. All behavioral experiments were performed in a controlled environment, and animals were acclimated for at least 30 min before testing.

To minimize fatigue, stress, and potential interference among behavioral paradigms, individual tests were conducted on separate days within each assessment period. Investigators performing behavioral assessments, histopathological evaluations, and neurochemical analyses were blinded to treatment allocation throughout data acquisition and analysis.

Because this study included exploratory mechanistic investigations in addition to behavioral assessments, some experiments were conducted using independent subgroups of animals. Consequently, sample sizes varied across individual behavioral, histopathological, and neurochemical analyses.

#### 2.3.1. Y-Maze Test

Spatial working memory was assessed using a Y-maze consisting of three arms positioned at 120° angles. Rats were allowed to explore freely for 8 min. An arm entry was defined as entry of all four paws. Spontaneous alternation was defined as consecutive entries into three different arms. The alternation rate (%) was calculated as:

Alternation rate = [number of alternations/(total arm entries − 2)] × 100

Total arm entries were recorded as an index of locomotor activity [26].

#### 2.3.2. Tape Removal Test

Sensorimotor function was evaluated using the adhesive removal test. A 6 × 6 mm adhesive tape was applied to the forepaw, and animals were observed for 180 s. The primary outcomes were success rate (%) of tape removal and number of attempts. Assessments were performed by two independent observers blinded to group allocation [27].

#### 2.3.3. Open Field Test

Locomotor activity and anxiety-like behavior were assessed in an open-field arena (53 × 53 × 40 cm). Rats were allowed to explore for 3 min. Total distance traveled and center occupancy ratio were recorded and analyzed using automated video tracking software [28,29].

#### 2.3.4. Grip Strength Test

Neuromuscular strength was measured using a digital grip strength meter (BIO-GS3, Bioseb, France). Each animal performed three consecutive trials, and the mean value was used for analysis. Forelimb and hindlimb grip strengths were assessed separately [30].

#### 2.3.5. Olfactory Discrimination Test

Olfactory function was assessed using a two-odor paradigm. Rats were exposed to familiar and novel odor stimuli and allowed to explore for 5 min. A discrimination index (DI) was calculated as:

DI = time spent in novel odor zone/total exploration time

The number of zone transitions was also recorded as an index of exploratory activity [31].

#### 2.3.6. Marble Burying Test

Rats were placed in cages containing bedding with 20 evenly spaced marbles. After 10 min, the number of buried marbles and digging events were recorded. A marble was considered buried if at least two-thirds of its surface was covered [32].

#### 2.3.7. Sucrose Preference Test

Hedonic behavior was assessed using a two-bottle choice paradigm. Rats were given free access to water and sucrose solution [33].

Sucrose preference (%) was calculated as:

Sucrose preference = [sucrose intake/(total fluid intake)] × 100

### 2.4. Neurochemical Analysis

Dopamine and serotonin concentrations in rat brain tissues were quantified using liquid chromatography–tandem mass spectrometry (LC–MS/MS). Neurochemical analyses were performed using tissue samples obtained from the left cerebral hemisphere. The cerebral cortex, hippocampus, basal ganglia, thalamus, and midbrain were rapidly dissected on ice and homogenized in 0.1% formic acid using a bead-based tissue homogenizer. Homogenates were centrifuged at 13,500 rpm for 5 min at 4 °C, and the resulting supernatants were collected for analysis.

For sample preparation, aliquots of tissue supernatants were mixed with an internal standard working solution containing dopamine-d4 and serotonin-d4, vortex-mixed, and centrifuged to remove residual particulates. The clarified supernatants were diluted with 0.1% formic acid in water and transferred to polypropylene autosampler vials for LC–MS/MS analysis.

Neurochemical quantification was performed using a SCIEX Triple Quad 6500 mass spectrometer (SCIEX, Framingham, MA, USA) operated in positive electrospray ionization mode with multiple reaction monitoring (MRM). Chromatographic separation was achieved on a Hypersil GOLD C18 column (2.1 × 150 mm, 1.9 μm; Thermo Fisher Scientific, Waltham, MA, USA) maintained at 40 °C. The mobile phases consisted of 0.1% formic acid in water (mobile phase A) and 0.1% formic acid in methanol (mobile phase B), delivered under gradient elution conditions.

Dopamine and serotonin concentrations were quantified using calibration curves generated with their respective deuterated internal standards. Dopamine concentrations were normalized to tissue weight and expressed as μg/g tissue, whereas serotonin concentrations were expressed as ng/g tissue. Neurochemical analyses were conducted in a predefined exploratory subgroup independent of the behavioral assessment cohort, and all sample analyses were performed with investigators blinded to treatment allocation.

### 2.5. Histopathology

Following euthanasia, brains were harvested, fixed in 10% neutral buffered formalin, embedded in paraffin, and sectioned at 4–5 μm thickness. Histopathological evaluation was performed on hematoxylin and eosin (H&E)-stained sections of the cortex, basal ganglia (striatum), hippocampal CA1 region, and thalamus by an independent pathology laboratory blinded to treatment allocation. Images were acquired using a light microscope equipped with a 20× objective lens and a 10× ocular lens, resulting in a total magnification of 200×.

Histopathological injury was assessed using a previously published semi-quantitative scoring system based on neuronal degeneration, edema, inflammatory cell infiltration, and tissue vacuolation. Additional histopathological features, including neuronal shrinkage and necrosis, were also evaluated. Each parameter was graded on a 5-point scale (0–4), and the total injury score was calculated as the sum of individual scores (maximum score: 20). For each animal, multiple representative microscopic fields from predefined anatomical regions were examined and incorporated into the final histopathological assessment.

Histopathological analysis was conducted as an exploratory mechanistic endpoint in a predefined subgroup of animals that was independent of the behavioral assessment cohort. In accordance with the Reduction principle of the 3Rs, the number of control animals allocated to histopathological analysis was intentionally minimized because substantial neuropathological abnormalities were not anticipated in non-exposed animals based on established neuropathological knowledge and pilot observations.

### 2.6. Statistical Analysis

Data are presented as mean ± standard deviation (SD). Given the relatively small sample sizes and the potential for non-normal data distribution, non-parametric statistical methods were used throughout the study. Between-group comparisons at individual time points were performed using the Mann–Whitney U test. Longitudinal within-group behavioral changes across baseline, 2 weeks, and 4 weeks were evaluated using the Friedman test followed by Dunn’s multiple-comparison test when appropriate. For comparisons involving more than two independent groups, the Kruskal–Wallis test with Dunn’s post hoc test was applied. Paired comparisons were analyzed using the Wilcoxon signed-rank test when applicable. Categorical variables, including tape-removal success rates, were analyzed using Fisher’s exact test. Correlations between variables were assessed using Spearman’s rank correlation coefficient.

A two-sided *p*-value < 0.05 was considered statistically significant. Sample sizes for individual experiments are indicated in the corresponding figures and tables.

Because this study was designed as an exploratory mechanistic study, formal a priori sample-size calculation was not performed. Sample sizes were determined based on pilot experiments and previous studies investigating delayed neurobehavioral alterations following acute carbon monoxide exposure.

Exclusion criteria were predefined before data analysis. No animals or data points were excluded unless technical failure occurred during behavioral testing or tissue processing; no such exclusions occurred in the present study.

## 3. Results

### 3.1. Survival, Exposure Optimization, and General Condition

Preliminary dose-finding experiments demonstrated 100% mortality at 10,000 ppm and near-complete mortality at 8000 ppm. At 6500 ppm, more than 90% of animals survived and exhibited measurable behavioral alterations, whereas at 6000 ppm, survival was complete, but fewer than 50% of animals showed detectable behavioral changes. Based on these findings, 6500 ppm for 25 min was selected as the optimal exposure condition.

Using this protocol, all animals survived CO exposure, and no acute mortality occurred during the study period. Although most CO-exposed animals exhibited transient lethargy and reduced spontaneous activity within the first 24 h after exposure, symptom severity varied among individuals and resolved thereafter. Humane endpoints were predefined before study initiation; however, no animals met euthanasia criteria during the experimental period.

Body weight did not differ significantly between the CO and control groups at baseline, 2 weeks, or 4 weeks after exposure (all *p* > 0.05), indicating comparable general health status throughout the study.

### 3.2. Y-Maze Test: Impairment of Spatial Working Memory

In the Y-maze test, spontaneous alternation rates were comparable between groups at baseline (Control, 69.5 ± 0.96%; CO, 69.3 ± 0.73%; *p* = 0.884). At 2 weeks after exposure, the CO-exposed group showed a significant reduction in spontaneous alternation compared with controls (59.1 ± 0.53% vs. 70.8 ± 0.94%; *p* < 0.0001), with a further decrease observed at 4 weeks (49.3 ± 0.47% vs. 72.2 ± 1.03%; *p* < 0.0001). Longitudinal analysis confirmed a progressive decline in spontaneous alternation in the CO-exposed group (Friedman *p* < 0.001), whereas no significant change was observed in controls. Total arm entries showed a modest decrease over time in CO-exposed rats.

### 3.3. Tape Removal Test: Sensorimotor Deficits

In the tape-removal test, removal latency, attempt counts, and success rates were comparable between groups at baseline. At 2 weeks after exposure, removal latency and attempt counts remained similar; however, the success rate was markedly reduced in the CO-exposed group compared with controls (11.1% vs. 100%). At 4 weeks, removal latency did not differ significantly between groups, whereas the number of removal attempts was significantly lower in the CO-exposed group (1.0 ± 0.42 vs. 3.2 ± 0.75; *p* = 0.041). Notably, none of the CO-exposed animals successfully removed the tape, whereas all control animals completed the task (0% vs. 100%). Longitudinal analysis demonstrated a significant deterioration in tape-removal performance over time in the CO-exposed group (*p* < 0.05), while performance remained stable in controls.

### 3.4. Open Field Test: Reduced Locomotor Activity

In the open field test, total distance traveled and center ratio were comparable between groups at baseline and 2 weeks after exposure. At 4 weeks, the CO-exposed group exhibited a significantly shorter total distance traveled than the control group (330.5 ± 172.1 cm vs. 730.5 ± 139.5 cm; *p* < 0.01). In contrast, center ratio did not differ significantly between groups at any time point.

Longitudinal analysis revealed significant changes in locomotor activity over time in both groups. Although repeated testing may have contributed to temporal variation, the reduction in total distance traveled was more pronounced in the CO-exposed group, indicating persistent locomotor impairment following CO exposure.

### 3.5. Olfactory Discrimination: Preserved Sensory Function

In the olfactory discrimination test, no significant differences were observed between groups in the discrimination index (DI) at any time point.

At baseline, a trend toward a lower DI was observed in the CO-exposed group compared with controls (19.5 vs. 36.2; *p* = 0.065), although this did not reach statistical significance. At 2 weeks (42.7 vs. 30.1; *p* = 0.545) and 4 weeks (47.8 vs. 45.2; *p* = 0.840), DI values remained comparable between groups.

### 3.6. Marble Burying: Reduced Exploratory Behavior

In the marble-burying test, digging behavior and the number of buried marbles were comparable between groups at baseline and 2 weeks after exposure. At 4 weeks, the CO-exposed group showed a significant reduction in digging behavior compared with controls (10.1 ± 6.9 vs. 27.4 ± 3.9; *p* < 0.01). The number of buried marbles was also significantly lower in the CO-exposed group than in controls (0.5 ± 1.1 vs. 4.4 ± 1.1; *p* < 0.01). Longitudinal analysis demonstrated a progressive decrease in the number of buried marbles in the CO-exposed group (*p* < 0.05), whereas no significant change was observed in controls.

### 3.7. Sucrose Preference Test: Preserved Reward Response

Sucrose preference did not differ significantly between groups at baseline (74.6 ± 10.1% vs. 72.3 ± 7.6%; *p* = 0.805), 2 weeks (80.7 ± 8.7% vs. 76.5 ± 10.2%; *p* = 0.277), or 4 weeks (82.3 ± 5.7% vs. 80.1 ± 11.4%; *p* = 0.895).

### 3.8. Grip Strength: Preserved Neuromuscular Function

Grip strength was assessed in both forelimbs and hindlimbs at baseline, 2 weeks, and 4 weeks.

No significant differences were observed between groups at any time point.

Forelimb grip strength was comparable between groups at baseline (1.85 ± 0.12 vs. 1.83 ± 0.10, *p* = 0.884), 2 weeks (2.10 ± 0.15 vs. 2.05 ± 0.13, *p* = 0.895), and 4 weeks (2.35 ± 0.18 vs. 2.30 ± 0.16, *p* = 0.854).

Similarly, hindlimb grip strength showed no significant group differences at baseline (3.2 ± 0.14 vs. 3.1 ± 0.15, *p* = 0.934), 2 weeks (3.7 ± 0.16 vs. 3.75 ± 0.18, *p* = 0.854), or 4 weeks (4.5 ± 0.20 vs. 4.6 ± 0.19, *p* = 0.882).

Overall, progressive deterioration was observed in several behavioral domains following CO poisoning, whereas olfactory discrimination, sucrose preference, and grip strength remained relatively preserved. Behavioral findings across all testing paradigms are summarized in Table 1. The functional interpretation of the major behavioral alterations is summarized in Table 2. Detailed results of the longitudinal within-group analyses are presented in Supplementary Table S1.

### 3.9. Neurochemical Analysis: Regional Monoaminergic Alterations

**Figure 2:**
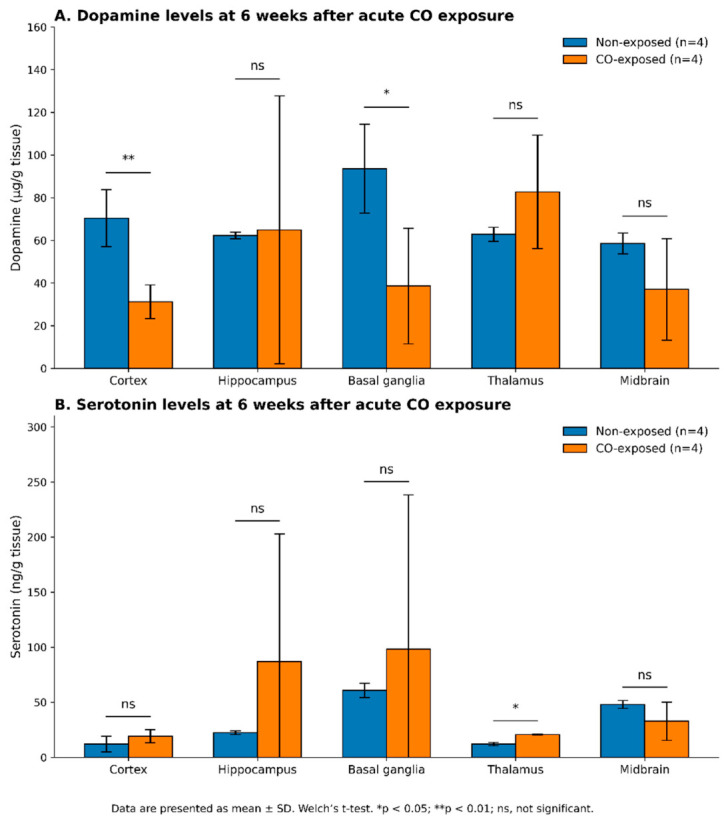
Dopamine and Serotonin levels in brain regions. Dopamine (**A**) and serotonin (**B**) concentrations were measured in the cortex, hippocampus, basal ganglia, thalamus, and midbrain of non-exposed and carbon monoxide (CO)-exposed rats at 6 weeks after acute CO exposure. Dopamine levels were significantly decreased in the cortex and basal ganglia of the CO-exposed group compared with controls, whereas serotonin levels were significantly increased in the thalamus following CO exposure. No significant differences were observed in the other brain regions. Data are presented as mean ± SD (*n* = 4 per group). Statistical analysis was performed using the Mann–Whitney U test. * *p* < 0.05; ** *p* < 0.01; ns, not significant.

Regional dopamine and serotonin concentrations are presented in Figure 2. Dopamine concentrations were significantly reduced in the cortex (31.18 ± 7.94 vs. 70.36 ± 13.30 ng/mL, *p* = 0.009) and basal ganglia (38.61 ± 27.12 vs. 93.60 ± 20.81 ng/mL, *p* = 0.048) of CO-exposed rats compared with controls. In contrast, thalamic serotonin levels were significantly elevated following CO exposure (20.58 ± 0.49 vs. 11.98 ± 1.41 ng/mL, *p* = 0.014), whereas other serotonergic changes did not reach statistical significance.

### 3.10. Histopathology: Increased Injury Score

**Figure 3:**
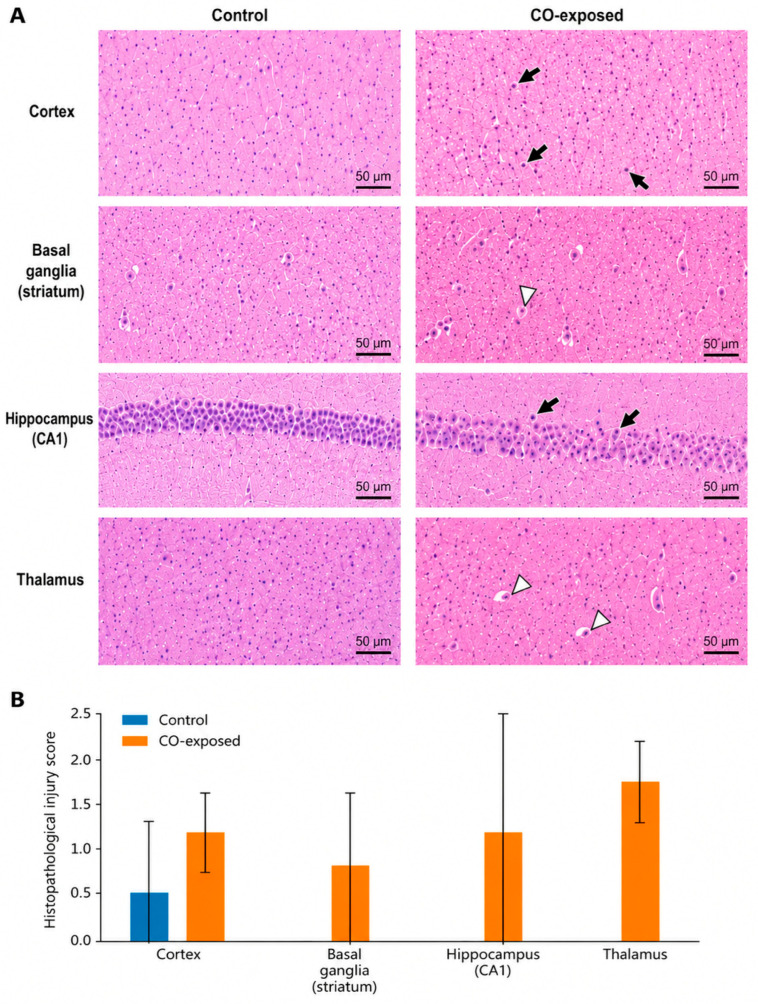
Histopathological alterations following acute carbon monoxide poisoning. (**A**) Representative hematoxylin and eosin (H&E)-stained sections of the cortex, basal ganglia (striatum), hippocampus (CA1), and thalamus obtained 6 weeks after CO poisoning. Representative H&E-stained sections of the cerebral cortex, basal ganglia (striatum), hippocampal CA1 region, and thalamus. Black arrows indicate shrunken neurons in the cerebral cortex and hippocampal CA1 region. White arrowheads indicate representative vacuolar changes in the basal ganglia (striatum) and thalamus. Images were obtained using a 20× objective lens and a 10× ocular lens (total magnification 200×). Scale bar = 50 μm. (**B**) Semi-quantitative regional histopathological injury scores. Histopathological injury was assessed using a blinded semi-quantitative scoring system based on neuronal degeneration, edema, inflammatory cell infiltration, neuronal shrinkage, and tissue vacuolation. The total histopathological injury score was calculated as the sum of individual parameter scores. Data are presented as mean ± SD (Control: *n* = 2; CO-exposed: *n* = 5).

The total histopathological score was significantly higher in the CO-exposed group compared with controls (5.5 ± 1.0 vs. 0.5 ± 0.7; *p* < 0.05). Representative histopathological findings and regional injury scores are shown in Figure 3.

## 4. Discussion

Behavioral impairments—including reduced alternation, locomotion, and diminished behavioral engagement—were accompanied by neuroinflammatory changes and alterations in monoaminergic profiles, suggesting a link between neuroinflammation, monoaminergic dysregulation, and functional deficits [34,35].

This study established a reproducible rat model that captures key behavioral, neurochemical, and histopathological features of DNS following acute CO poisoning. Reproducible experimental models faithfully reflecting the delayed and progressive nature of DNS have remained limited. Using a controlled exposure paradigm, we induced consistent delayed impairments across multiple functional domains, including cognition, locomotion, sensorimotor integration, anxiety-related behavior, and behavioral engagement, accompanied by persistent histopathological alterations and region-specific changes in monoaminergic signaling. These findings support the concept that DNS represents an active and evolving neuropathological process rather than a static consequence of transient hypoxic injury [2,9].

The behavioral phenotype observed in this model closely parallels clinical manifestations of DNS, including progressive working memory impairment, psychomotor slowing, reduced exploratory activity, and impaired behavioral engagement (Table 1) [5,36]. Importantly, these abnormalities emerged after a latent period and worsened over time, enhancing the translational relevance of the model. Consistent with this pattern, longitudinal analyses demonstrated progressive declines in spatial working memory, sensorimotor function, and exploratory behavior in CO-exposed rats, whereas control animals remained largely stable across behavioral domains (Supplementary Table S1). Notably, Y-maze performance deteriorated continuously from baseline to 4 weeks, indicating that delayed cognitive dysfunction evolves progressively rather than representing a static residual deficit following acute poisoning. These findings support the concept that DNS is an active and evolving neuropathological process and underscore the robustness of this experimental model.

Sensorimotor deficits demonstrated by the tape removal test were particularly notable. Although gross motor strength remained preserved, CO-exposed rats showed markedly reduced task engagement and success rates, suggesting dysfunction in sensory integration, motor initiation, attentional processing, or motivational circuits rather than simple peripheral motor weakness. This interpretation is supported by preserved forelimb and hindlimb grip strength, indicating intact peripheral neuromuscular function. Together, these findings point toward dysfunction within central neural circuits rather than peripheral systems.

The open field test demonstrated reduced locomotor activity in CO-exposed animals, suggesting persistent impairment of spontaneous exploratory movement. However, no significant differences were observed in center occupancy. Similarly, marble burying behavior was significantly reduced, suggesting impaired exploratory drive and reduced motivated behavior rather than enhanced compulsive behavior. Given that these behaviors are strongly modulated by cortico-striatal and limbic circuitry, these findings suggest persistent dysfunction within distributed neural networks involved in behavioral regulation [37,38]. Interestingly, longitudinal analysis demonstrated temporal changes in locomotor activity in both groups. This observation suggests that repeated exposure to the open-field environment and habituation effects may partially contribute to reductions in exploratory locomotion over time. Nevertheless, the magnitude of decline was substantially greater in CO-exposed animals, supporting the presence of additional CO-related locomotor dysfunction beyond normal adaptation to repeated testing.

In contrast, sucrose preference remained relatively preserved throughout the observation period. Although control animals exhibited a gradual increase in sucrose preference over time, this adaptive increase appeared attenuated in CO-exposed rats. While subtle, this divergence may reflect impaired reward-related behavioral adaptation rather than complete loss of reward responsiveness. Olfactory discrimination also remained preserved across time points, suggesting relative resistance of primary olfactory pathways to CO-induced injury or the presence of compensatory mechanisms maintaining olfactory function [39]. Collectively, these findings indicate domain-specific vulnerability following CO exposure, in which cognitive, sensorimotor, and exploratory behaviors are preferentially affected while certain primary sensory and hedonic functions remain relatively preserved (Table 1) [40,41].

A central finding of this study is the presence of delayed-phase dopaminergic alteration. Neurochemical analysis demonstrated significantly reduced dopamine levels in the cortex and basal ganglia of CO-exposed rats. Given the established role of cortical and striatal dopaminergic signaling in executive function, attention, working memory, motivation, locomotion, and motor coordination, these alterations provide a plausible mechanistic link between CO exposure and the multidomain behavioral impairments observed in this model [21,30,42]. Importantly, although statistically significant reductions were observed primarily in the cortex and basal ganglia, dopamine levels showed a general downward trend across multiple brain regions in CO-exposed animals. Considering the limited sample size and variability inherent to neurochemical measurements, the absence of statistical significance in certain regions should be interpreted with caution. It is therefore possible that CO-induced dopaminergic dysfunction extends beyond focal changes and reflects a broader system-level disturbance affecting distributed neural circuits [18,34,35]. The observed cortical and basal ganglia dopamine depletion is consistent with previous evidence suggesting that CO poisoning may disrupt dopaminergic neurotransmission and contribute to delayed neurobehavioral dysfunction.

**Figure 4:**
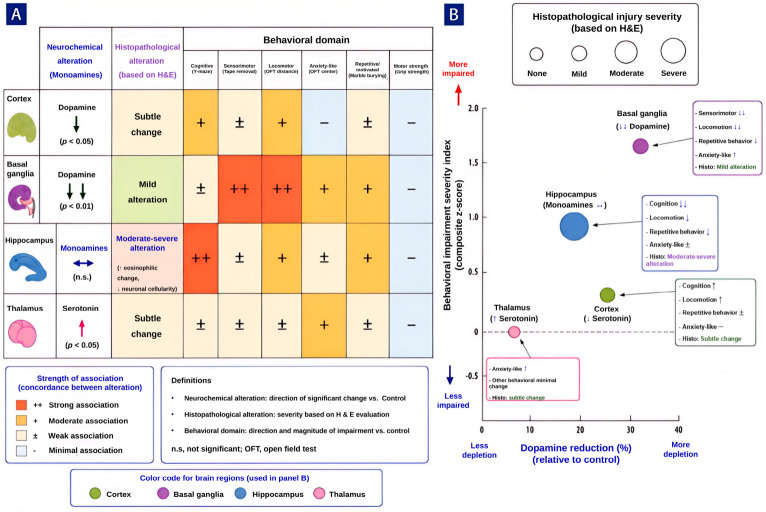
Summary of delayed behavioral, histopathological, and neurochemical alterations following acute carbon monoxide poisoning. (**A**) Heatmap summarizing the relative concordance among regional neurochemical alterations, histopathological findings, and behavioral abnormalities following acute carbon monoxide (CO) poisoning. Behavioral outcomes were derived from assessments performed 4 weeks after CO poisoning, whereas neurotransmitter and histopathological analyses were obtained at 6 weeks after exposure. (**B**) Bubble severity plot illustrating the relative relationship among regional dopamine depletion, behavioral impairment severity, and histopathological burden. Bubble size represents relative histopathological injury severity based on H&E evaluation. The basal ganglia demonstrated the strongest overall concordance among dopamine depletion, histopathological injury, and behavioral abnormalities, particularly in sensorimotor and locomotor domains. Cortical dopamine depletion was observed in parallel with impaired locomotor and cognitive performance despite only subtle histopathological alterations, suggesting that neurochemical dysfunction may occur in the absence of overt structural injury following CO exposure. The hippocampus exhibited prominent behavioral and histopathological abnormalities despite minimal monoaminergic alterations, indicating that delayed CO-induced neurotoxicity is likely mediated by multiple mechanisms beyond monoaminergic dysregulation alone. Thalamic serotonin elevation may reflect region-specific serotonergic alterations, although their behavioral significance remains uncertain. Gross motor strength remained preserved despite persistent neurochemical and histopathological abnormalities. Histopathological and neurochemical analyses were performed in partially independent animal subgroups. Therefore, relationships illustrated in this figure represent exploratory concordance across experimental domains and should not be interpreted as direct animal-level correlations or causal associations.

The integrated analysis of behavioral, neurochemical, and histopathological findings summarized in Figure 4 further supports this interpretation. Basal ganglia showed the strongest concordance between dopamine depletion, histopathological injury, and behavioral impairment, particularly in sensorimotor and locomotor domains. In contrast, cortical dopamine depletion was associated with impaired locomotor and cognitive performance despite only subtle histopathological alterations, suggesting that neurochemical dysfunction may precede overt structural injury following CO exposure. These findings collectively support the possibility that delayed monoaminergic dysregulation contributes to persistent circuit dysfunction in DNS [43,44]. However, because histopathological and neurochemical analyses were performed in partially independent animal subgroups, these relationships should be interpreted as exploratory concordance across experimental domains rather than direct animal-level correlations.

Interestingly, the hippocampus exhibited marked behavioral and histopathological abnormalities despite minimal monoamine alteration. Histopathological examination demonstrated persistent eosinophilic neuronal change and reduced neuronal cellularity within hippocampal regions, whereas monoaminergic alterations were relatively limited. This dissociation is visually summarized in Figure 4 and suggests that delayed CO-induced neurotoxicity cannot be explained solely by monoaminergic depletion and may involve additional mechanisms such as neuroinflammation, synaptic dysfunction, mitochondrial injury, or impaired network connectivity [40,45].

The present findings also suggest a dynamic neurochemical trajectory following CO exposure. Previous studies have reported transient increases in dopamine during the acute phase after CO poisoning [12,19,46,47], whereas the current study demonstrated delayed reductions during the chronic phase. This pattern supports a biphasic response in which early compensatory neurotransmitter release is followed by delayed depletion, potentially driven by neuroinflammatory processes, impaired neurotransmitter synthesis, oxidative stress, or progressive circuit remodeling [18,42,48,49]. Within this framework, delayed dopaminergic dysfunction may represent a key contributor to persistent neurological impairment in DNS.

Serotonergic alterations appeared less pronounced than dopaminergic changes in the present model. Although no statistically significant differences were observed in serotonin levels across most regions, a tendency toward increased serotonin concentrations was observed in several brain regions of CO-exposed rats. In particular, increased thalamic serotonin levels were observed following CO exposure. However, the behavioral significance of this finding remains uncertain because no significant differences were observed in center occupancy or other anxiety-related behavioral measures. Given the known role of serotonin in mood regulation, stress responsiveness, and behavioral modulation, even modest serotonergic alterations may influence network-level behavioral outcomes through interaction with dopaminergic systems [50].

However, these serotonergic findings should be interpreted cautiously. Variability among animals and the relatively small sample size may have limited the ability to detect subtle but biologically meaningful changes. In addition, serotonergic alterations may occur in a region-specific or time-dependent manner not fully captured by the present experimental design. Further studies incorporating larger cohorts and longitudinal neurochemical analyses will be necessary to clarify the contribution of serotonergic pathways to delayed CO-induced neurobehavioral dysfunction.

Histopathological analysis revealed mild but persistent structural alterations, including eosinophilic neuronal change, neuronal shrinkage, reduced neuronal cellularity, and inflammatory cell infiltration, particularly within the hippocampus and basal ganglia. Although the overall degree of structural injury was relatively modest, the persistence of these abnormalities several weeks after exposure suggests ongoing delayed neuropathological processes rather than transient acute injury alone. The convergence of behavioral impairment, monoaminergic dysregulation, and persistent histopathological change supports the concept that delayed neuroinflammatory and circuit-level dysfunction contribute substantially to DNS pathogenesis [9,51].

Importantly, gross motor strength remained preserved despite persistent neurochemical and structural alterations. This dissociation further supports the concept that delayed CO-induced neurotoxicity preferentially affects higher-order neural circuits involved in cognition, behavioral regulation, sensorimotor integration, and motivation rather than basic peripheral motor output systems [52,53].

A major strength of this study is the establishment of a reproducible experimental model that captures the delayed and progressive characteristics of DNS. Unlike previous models focused primarily on acute toxicity, this model enables systematic investigation of delayed functional impairment and associated neurochemical and histopathological mechanisms. This provides a potentially valuable platform for mechanistic studies and therapeutic development targeting delayed CO-induced brain injury.

Several limitations should be acknowledged. First, the sample size was relatively small, potentially limiting statistical power to detect subtle neurochemical differences. In particular, histopathological analyses were intentionally performed in a limited number of animals as an exploratory mechanistic assessment in accordance with the Reduction principle of the 3Rs. Consequently, statistical interpretation of regional injury severity is limited, and these findings should be considered exploratory pending confirmation in larger cohorts. Second, neurochemical analyses were primarily limited to dopamine and serotonin, whereas additional neurotransmitter systems—including glutamatergic, GABAergic, and cholinergic pathways—may also contribute to the pathophysiology of delayed neuropsychiatric sequelae (DNS). Third, histopathological evaluation was based on a semi-quantitative scoring system, which may have limited sensitivity for detecting subtle regional injury patterns. In addition, histopathological assessment was performed in a limited number of animals. Therefore, these histopathological findings should be interpreted as supportive evidence and considered in conjunction with the biochemical and functional outcomes of the study. Future studies incorporating quantitative inflammatory and neuronal injury markers may provide additional insight into the mechanisms underlying delayed neurological dysfunction. Fourth, although behavioral assessments within each paradigm were performed longitudinally in the same animals, histopathological and neurochemical analyses were conducted in partially independent subgroups. Consequently, relationships among behavioral abnormalities, regional monoaminergic alterations, and histopathological findings should be interpreted as exploratory concordance rather than direct animal-level associations. Finally, although this model reproduces several key features of DNS, it may not fully capture the clinical heterogeneity observed in human carbon monoxide poisoning.

Despite these limitations, the longitudinal behavioral analyses (Supplementary Table S1) consistently demonstrated progressive deterioration across multiple behavioral domains, supporting the robustness of the observed DNS phenotype.

## 5. Conclusions

Acute CO poisoning induced a reproducible DNS characterized by progressive multidomain behavioral impairment, persistent histopathological abnormalities, and region-specific monoaminergic dysregulation. Cortical and basal ganglia dopaminergic dysfunction showed partial concordance with cognitive, sensorimotor, and locomotor deficits, suggesting that delayed disruption of dopaminergic circuits may contribute to the evolving pathophysiology of DNS. However, the dissociation between prominent hippocampal injury and limited monoaminergic alteration indicates that delayed CO-induced neurotoxicity is not mediated by monoaminergic depletion alone, but likely reflects multiple interacting mechanisms, including neuroinflammation and circuit-level dysfunction. Collectively, these findings establish a reproducible experimental model of DNS, provide an integrated framework for understanding delayed CO-induced brain injury, and support future therapeutic strategies targeting neuroinflammatory and dopaminergic pathways.

## Figures and Tables

**Table 1:** Behavioral test outcomes.

Test	Time	Control (Mean ± SD)	CO-Exposed (Mean ± SD)	*p*-Value
Y-maze	Pre	69.5 ± 0.96	69.3 ± 0.73	0.884
	2 w	70.8 ± 0.94	59.1 ± 0.53	<0.0001
	4 w	72.2 ± 1.03	49.3 ± 0.47	<0.0001
Tape removal (attempts)	Pre	5.9 ± 2.80	5.8 ± 1.55	0.954
	2 w	5.8 ± 3.40	5.3 ± 2.14	0.784
	4 w	3.4 ± 1.82	1.0 ± 0.94	0.033
Tape removal (success, %)	Pre	100	100	1.00
	2 w	100	12.5	<0.05
	4 w	100	0	<0.001
OFT (distances, cm)	Pre	1119.9 ± 210.2	954.1 ± 471.4	0.537
	2 w	676.8 ± 182.9	601.0 ± 271.6	0.792
	4 w	730.5 ± 139.5	330.5 ± 172.1	<0.05
OFT (central occupancy ratio)	Pre	0.05 ± 0.02	0.07 ± 0.05	0.855
	2 w	0.06 ± 0.05	0.10 ± 0.07	0.464
	4 w	0.15 ± 0.11	0.10 ± 0.09	0.314
ODT (DI)	Pre	36.2	30.4	0.685
	2 w	30.1	42.7	0.545
	4 w	45.2	47.8	0.840
ODT (Number of transitions)	Pre	19.4	19.5	0.792
	2 w	15.5	10.7	0.185
	4 w	10.6	8.8	0.153
MBT (buried marbles)	Pre	6.8 ± 4.0	5.6 ± 4.8	0.833
	2 w	6.0 ± 2.0	6.5 ± 4.3	0.881
	4 w	4.4 ± 1.1	0.5 ± 1.1	0.003
MBT (Number of digging)	Pre	33.0 ± 8.3	21.6 ± 17.3	0.463
	2 w	18.8 ± 6.7	22.9 ± 19.1	1.000
	4 w	27.4 ± 3.9	10.1 ± 6.9	0.004
SPT (preference, %)	Pre	74.6 ± 10.1	72.3 ± 7.6	0.805
	2 w	80.7 ± 8.7	76.5 ± 10.2	0.277
	4 w	82.3 ± 5.7	80.1 ± 11.4	0.895
Grip strength (forefoot, N)	Pre	1.85 ± 0.12	1.83 ± 0.10	0.884
	2 w	2.10 ± 0.15	2.05 ± 0.13	0.895
	4 w	2.35 ± 0.18	2.30 ± 0.16	0.854
Grip strength (hindfoot, N)	Pre	3.2 ± 0.14	3.1 ± 0.15	0.934
	2 w	3.7 ± 0.16	3.75 ± 0.18	0.854
	4 w	4.5 ± 0.20	4.6 ± 0.19	0.882

Data are presented as mean ± SD. Sample sizes varied by experiment: Y-maze (Control *n* = 8, CO *n* = 8), tape removal (Control *n* = 5, CO *n* = 8), open field (Control *n* = 6, CO *n* = 6), olfactory discrimination (Control *n* = 7, CO *n* = 7), marble burying (Control *n* = 6, CO *n* = 6), sucrose preference (Control *n* = 5, CO *n* = 5), and grip strength (Control *n* = 8, CO *n* = 8).

**Table 2:** Functional interpretation of significant behavioral alterations following acute CO poisoning.

Behavioral Test	Functional Domain	Effect of CO Exposure	Interpretation
**Y-maze**	Spatial working memory	Significantly impaired	Delayed cognitive dysfunction involving hippocampal-dependent working memory
**Tape removal test**	Sensorimotor integration and somatosensory function	Markedly impaired	Persistent impairment in sensorimotor processing and coordinated task performance
**Open field test (distance traveled)**	Spontaneous locomotor and exploratory activity	Significantly reduced	Decreased exploratory behavior and locomotor activity
**Olfactory discrimination test**	Olfactory recognition and discrimination	No significant impairment	Preserved olfactory cognitive function
**Marble burying test**	Innate digging and repetitive behavior	Significantly reduced	Impaired motivated and goal-directed exploratory behavior
**Sucrose preference test**	Hedonic behavior	No significant impairment	No evidence of anhedonia or depressive-like behavior
**Grip strength test (forelimb/hindlimb)**	Gross motor strength	No significant impairment	Preserved muscular strength despite delayed neurotoxicity
**Behavioral test**	Functional domain	Effect of CO exposure	Interpretation
**Y-maze**	Spatial working memory	Significantly impaired	Delayed cognitive dysfunction involving hippocampal-dependent working memory
**Tape removal test**	Sensorimotor integration and somatosensory function	Markedly impaired	Persistent impairment in sensorimotor processing and coordinated task performance
**Open field test (distance traveled)**	Spontaneous locomotor and exploratory activity	Significantly reduced	Decreased exploratory behavior and locomotor activity
**Olfactory discrimination test**	Olfactory recognition and discrimination	No significant impairment	Preserved olfactory cognitive function
**Marble burying test**	Innate digging and repetitive behavior	Significantly reduced	Impaired motivated and goal-directed exploratory behavior
**Sucrose preference test**	Hedonic behavior	No significant impairment	No evidence of anhedonia or depressive-like behavior
**Grip strength test (forelimb/hindlimb)**	Gross motor strength	No significant impairment	Preserved muscular strength despite delayed neurotoxicity

## Data Availability

The data presented in this study are available from the corresponding author upon reasonable request.

## Supplementary Materials

The following supporting information can be downloaded at: https://www.mdpi.com/article/10.3390/brainsci16060647/s1, Table S1: Longitudinal within-group behavioral changes evaluated using the Friedman test.
