# Supplementary material for: Progressive Behavioral Impairment and Region-Specific Monoaminergic Alterations in a Rat Model of Delayed Neuropsychiatric Sequelae After Acute Carbon Monoxide Poisoning
Source: Brain Sci. 2026 Jun 18;16(6):647. doi: 10.3390/brainsci16060647 (PMC13297533; doi:10.3390/brainsci16060647)
Supplement: Supplementary file 1 [file brainsci-16-00647-s001.zip › brainsci-4339528-supplementary.pdf]

Supplementary Table S1. Longitudinal within-group behavioral changes evaluated using the Friedman test.

Test	Group	Pre	2w	4w	Friedman *p*-value	Post hoc comparison
**Y-maze**	Control	69.5 ± 0.96	70.8 ± 0.94	72.2 ± 1.03	0.887	ns
	CO-exposed	69.3 ± 0.73	59.1 ± 0.53	49.3 ± 0.47	<0.001	Pre vs 2w, Pre vs 4w, 2w vs 4w
**Tape removal attempts**	Control	5.9 ± 2.80	5.8 ± 3.40	3.4 ± 1.82	0.654	ns
	CO-exposed	5.8 ± 1.55	5.3 ± 2.14	1.0 ± 0.94	<0.05	Pre vs 4w
**OFT distance**	Control	1119.9 ± 210.2	676.8 ± 182.9	730.5 ± 139.5	<0.05	Pre vs 2w or ns
	CO-exposed	954.1 ± 471.4	601.0 ± 271.6	330.5 ± 172.1	<0.05	Pre vs 4w
**MBT buried marbles**	Control	6.8 ± 4.0	6.0 ± 2.0	4.4 ± 1.1	0.688	ns
	CO-exposed	5.6 ± 4.8	6.5 ± 4.3	0.5 ± 1.1	<0.05	2w vs 4w
